# Supplementary material for: Fibroblast and keratinocyte gene expression following exposure to extracts of neem plant (Azadirachta indica)
Source: Data Brief. 2017 Dec 20;16:982–92. doi: 10.1016/j.dib.2017.12.035 (PMC5752095; doi:10.1016/j.dib.2017.12.035)
Supplement: Supplementary file 2 — Supplementary material [file mmc2.docx]

Supplemental data 1. Ct value average ($\pm$SE) in Figure.1

|  |  |  | 0.001% | | | | | | | | 0.01% | | | | | | | |
| --- | --- | --- | --- | --- | --- | --- | --- | --- | --- | --- | --- | --- | --- | --- | --- | --- | --- | --- |
|  | control(0h) | | 2h | | 4h | | 8h | | 24h | | 2h | | 4h | | 8h | | 24h | |
| GAPDH | 15.11 | (±0.15) | 15.04 | (±0.14) | 14.78 | (±0.06) | 15.01 | (±0.27) | 15.03 | (±0.17) | 14.89 | (±0.12) | 14.72 | (±0.14) | 14.83 | (±0.11) | 15.07 | (±0.14) |
| HAS1 | 24.76 | (±0.22) | 26.73 | (±0.38) | 27.41 | (±0.27) | 27.63 | (±0.33) | 27.85 | (±0.47) | 26.51 | (±0.20) | 28.63 | (±0.22) | 27.59 | (±0.38) | 27.86 | (±0.50) |
| HAS2 | 20.45 | (±0.22) | 20.52 | (±0.19) | 20.40 | (±0.17) | 20.96 | (±0.28) | 21.42 | (±0.14) | 20.46 | (±0.23) | 20.79 | (±0.16) | 21.69 | (±0.38) | 21.71 | (±0.14) |
| HyAL1 | 27.26 | (±0.14) | 27.11 | (±0.23) | 26.96 | (±0.17) | 26.71 | (±0.14) | 26.55 | (±0.16) | 27.39 | (±0.22) | 27.52 | (±0.12) | 26.90 | (±0.22) | 26.75 | (±0.18) |
| HyAL2 | 22.74 | (±0.26) | 22.55 | (±0.25) | 23.02 | (±0.25) | 23.46 | (±0.41) | 23.21 | (±0.32) | 22.87 | (±0.17) | 22.93 | (±0.26) | 23.29 | (±0.25) | 23.14 | (±0.27) |
| V3 | 27.58 | (±0.14) | 27.53 | (±0.28) | 27.76 | (±0.14) | 28.26 | (±0.23) | 27.83 | (±0.25) | 27.86 | (±0.22) | 27.97 | (±0.17) | 28.14 | (±0.24) | 27.58 | (±0.14) |
| aggrecan | 25.82 | (±0.18) | 25.48 | (±0.41) | 25.41 | (±0.28) | 25.70 | (±0.25) | 25.90 | (±0.27) | 25.58 | (±0.23) | 25.58 | (±0.17) | 25.91 | (±0.37) | 26.40 | (±0.30) |
| CD44 | 18.31 | (±0.17) | 17.72 | (±0.09) | 17.75 | (±0.03) | 17.75 | (±0.07) | 18.25 | (±0.06) | 17.84 | (±0.07) | 17.90 | (±0.06) | 17.92 | (±0.05) | 18.27 | (±0.11) |
| COL1A1 | 14.91 | (±0.24) | 14.27 | (±0.20) | 13.98 | (±0.11) | 13.80 | (±0.05) | 13.39 | (±0.03) | 14.75 | (±0.13) | 14.11 | (±0.11) | 13.93 | (±0.12) | 13.46 | (±0.04) |
| COL3A1 | 16.99 | (±0.19) | 16.48 | (±0.13) | 16.17 | (±0.05) | 16.09 | (±0.07) | 15.41 | (±0.06) | 16.74 | (±0.04) | 16.61 | (±0.08) | 16.20 | (±0.11) | 15.67 | (±0.04) |
| COL7A1 | 23.78 | (±0.29) | 23.34 | (±0.23) | 23.25 | (±0.17) | 23.25 | (±0.13) | 23.81 | (±0.07) | 23.97 | (±0.18) | 23.76 | (±0.17) | 23.40 | (±0.10) | 23.78 | (±0.08) |
| MMP1 | 17.16 | (±0.19) | 16.92 | (±0.04) | 17.10 | (±0.02) | 17.36 | (±0.13) | 19.17 | (±0.07) | 17.00 | (±0.03) | 17.35 | (±0.07) | 17.46 | (±0.03) | 19.25 | (±0.05) |
| acid ceramidase | 21.45 | (±0.21) | 20.95 | (±0.04) | 20.95 | (±0.02) | 20.75 | (±0.11) | 20.80 | (±0.05) | 21.10 | (±0.01) | 21.33 | (±0.08) | 21.07 | (±0.07) | 20.81 | (±0.09) |
| bFGF | 19.10 | (±0.41) | 18.49 | (±0.32) | 19.29 | (±0.20) | 19.44 | (±0.18) | 18.75 | (±0.14) | 18.84 | (±0.36) | 20.59 | (±0.33) | 20.40 | (±0.23) | 19.34 | (±0.22) |
| FGF7 | 23.29 | (±0.50) | 22.63 | (±0.30) | 22.49 | (±0.16) | 22.57 | (±0.19) | 22.53 | (±0.15) | 22.42 | (±0.12) | 23.62 | (±0.15) | 23.26 | (±0.23) | 22.83 | (±0.26) |
| VEGF | 25.48 | (±0.20) | 24.32 | (±0.18) | 24.86 | (±0.13) | 24.44 | (±0.20) | 23.45 | (±0.07) | 24.30 | (±0.28) | 25.18 | (±0.05) | 24.91 | (±0.15) | 23.75 | (±0.14) |
| IL-1a | 26.55 | (±0.15) | 26.18 | (±0.11) | 26.53 | (±0.14) | 26.58 | (±0.12) | 26.44 | (±0.14) | 27.11 | (±0.06) | 26.94 | (±0.12) | 26.84 | (±0.25) | 26.39 | (±0.16) |
| cox2 | 22.65 | (±0.18) | 21.90 | (±0.27) | 23.23 | (±0.20) | 23.43 | (±0.39) | 23.66 | (±0.08) | 21.83 | (±0.51) | 23.40 | (±0.32) | 23.89 | (±0.33) | 23.94 | (±0.10) |
| TGFb | 20.18 | (±0.30) | 19.58 | (±0.26) | 19.70 | (±0.13) | 19.97 | (±0.15) | 20.89 | (±0.19) | 19.69 | (±0.28) | 19.60 | (±0.16) | 19.81 | (±0.11) | 20.85 | (±0.22) |
| AQP3 | 30.39 | (±0.57) | 30.68 | (±0.77) | 30.30 | (±0.82) | 29.97 | (±0.91) | 30.92 | (±1.00) | 30.48 | (±0.86) | 30.27 | (±0.87) | 30.13 | (±0.76) | 30.95 | (±1.00) |

Supplemental data 2. Ct value average ($\pm$SE) in Figure.2

|  |  |  | 0.001% | | | | | | | | 0.01% | | | | | | | |
| --- | --- | --- | --- | --- | --- | --- | --- | --- | --- | --- | --- | --- | --- | --- | --- | --- | --- | --- |
|  | control(0h) | | 2h | | 4h | | 8h | | 24h | | 2h | | 4h | | 8h | | 24h | |
| GAPDH | 16.78 | (±0.35) | 17.40 | (±0.50) | 17.11 | (±0.47) | 17.54 | (±0.51) | 17.51 | (±0.47) | 17.49 | (±0.48) | 17.28 | (±0.50) | 17.22 | (±0.47) | 17.51 | (±0.44) |
| HAS1 | 33.36 | (±0.23) | 32.74 | (±0.30) | 32.60 | (±0.28) | 32.77 | (±0.17) | 32.20 | (±0.20) | 32.60 | (±0.26) | 33.17 | (±0.33) | 32.83 | (±0.24) | 32.26 | (±0.22) |
| HAS2 | 27.44 | (±0.06) | 28.45 | (±0.19) | 27.30 | (±0.19) | 28.05 | (±0.13) | 28.51 | (±0.10) | 27.64 | (±0.24) | 27.94 | (±0.31) | 27.38 | (±0.26) | 28.24 | (±0.37) |
| HyAL1 | 26.56 | (±0.07) | 25.88 | (±0.41) | 25.78 | (±0.38) | 26.66 | (±0.05) | 26.50 | (±0.06) | 26.63 | (±0.12) | 26.82 | (±0.05) | 26.61 | (±0.06) | 26.55 | (±0.07) |
| HyAL2 | 21.80 | (±0.06) | 22.08 | (±0.11) | 22.09 | (±0.04) | 22.32 | (±0.03) | 21.84 | (±0.04) | 22.41 | (±0.27) | 22.13 | (±0.10) | 22.27 | (±0.07) | 21.74 | (±0.11) |
| V3 | 31.62 | (±0.12) | 31.22 | (±0.15) | 31.40 | (±0.23) | 32.25 | (±0.15) | 31.59 | (±0.13) | 31.78 | (±0.20) | 31.61 | (±0.20) | 31.64 | (±0.08) | 31.14 | (±0.09) |
| CD44 | 18.39 | (±0.07) | 18.45 | (±0.12) | 18.59 | (±0.12) | 18.76 | (±0.04) | 18.59 | (±0.02) | 18.75 | (±0.25) | 18.51 | (±0.06) | 18.67 | (±0.05) | 18.45 | (±0.09) |
| IL-1a | 26.23 | (±0.10) | 26.18 | (±0.06) | 26.59 | (±0.08) | 26.20 | (±0.10) | 26.12 | (±0.14) | 26.91 | (±0.40) | 26.60 | (±0.25) | 26.05 | (±0.16) | 26.19 | (±0.08) |
| cox2 | 21.19 | (±0.06) | 21.90 | (±0.13) | 21.04 | (±0.05) | 20.46 | (±0.06) | 21.22 | (±0.07) | 21.96 | (±0.34) | 21.19 | (±0.02) | 20.88 | (±0.05) | 21.37 | (±0.07) |
| TGFb | 20.60 | (±0.07) | 20.65 | (±0.08) | 20.72 | (±0.04) | 20.99 | (±0.03) | 20.72 | (±0.03) | 20.74 | (±0.05) | 20.69 | (±0.07) | 20.96 | (±0.04) | 20.66 | (±0.10) |
| AQP3 | 24.00 | (±0.10) | 24.87 | (±0.05) | 24.64 | (±0.06) | 25.18 | (±0.17) | 23.94 | (±0.09) | 25.15 | (±0.16) | 24.99 | (±0.14) | 25.51 | (±0.08) | 24.16 | (±0.09) |
| Laminin5 | 22.89 | (±0.12) | 22.66 | (±0.05) | 22.83 | (±0.09) | 22.77 | (±0.05) | 22.37 | (±0.03) | 23.37 | (±0.30) | 22.74 | (±0.08) | 22.76 | (±0.08) | 22.28 | (±0.06) |
| COL17A1 | 18.31 | (±0.07) | 18.03 | (±0.05) | 18.21 | (±0.04) | 18.28 | (±0.05) | 18.02 | (±0.02) | 18.75 | (±0.24) | 18.17 | (±0.07) | 18.24 | (±0.08) | 18.01 | (±0.11) |
| ITGA6 | 16.79 | (±0.06) | 16.89 | (±0.10) | 16.99 | (±0.07) | 17.07 | (±0.05) | 17.04 | (±0.07) | 17.38 | (±0.44) | 17.01 | (±0.07) | 16.91 | (±0.13) | 16.93 | (±0.13) |
| CRES3 | 28.93 | (±0.09) | 29.13 | (±0.11) | 28.99 | (±0.11) | 28.91 | (±0.11) | 28.64 | (±0.14) | 28.90 | (±0.09) | 28.93 | (±0.07) | 28.76 | (±0.08) | 28.68 | (±0.07) |
| ELOVL1 | 21.66 | (±0.04) | 22.19 | (±0.07) | 22.03 | (±0.08) | 22.28 | (±0.09) | 21.93 | (±0.06) | 22.26 | (±0.18) | 22.03 | (±0.05) | 22.11 | (±0.01) | 21.89 | (±0.13) |
| ELOVL4 | 23.35 | (±0.06) | 23.47 | (±0.09) | 23.53 | (±0.02) | 23.42 | (±0.04) | 22.88 | (±0.04) | 23.72 | (±0.27) | 23.44 | (±0.06) | 23.07 | (±0.04) | 22.75 | (±0.09) |
| FLG | 28.39 | (±0.13) | 28.41 | (±0.18) | 27.58 | (±0.18) | 28.19 | (±0.21) | 25.86 | (±0.12) | 27.96 | (±0.21) | 27.63 | (±0.05) | 27.06 | (±0.07) | 25.78 | (±0.09) |
| TGM1 | 24.68 | (±0.07) | 25.15 | (±0.07) | 24.88 | (±0.05) | 25.08 | (±0.04) | 24.24 | (±0.11) | 25.40 | (±0.24) | 25.08 | (±0.05) | 24.91 | (±0.06) | 24.32 | (±0.10) |
| KRT1 | 23.87 | (±0.09) | 23.68 | (±0.08) | 23.20 | (±0.12) | 22.73 | (±0.01) | 21.15 | (±0.10) | 24.13 | (±0.26) | 23.24 | (±0.10) | 22.62 | (±0.11) | 21.09 | (±0.10) |
